# Strength Level of Professional Elite Soccer Players after the COVID-19 Lockdown Period: A Retrospective Double-Arm Cohort Study

**DOI:** 10.1155/2022/8242210

**Published:** 2022-03-03

**Authors:** Robson Dias Scoz, Ricardo Lima Burigo, Isabella Christina Ferreira, Luiz Hespanhol, Ana Paula Silveira Ramos, Adriano Schlosser, Jose Joao Baltazar Mendes, Luciano Maia Alves Ferreira, César Ferreira Amorim

**Affiliations:** ^1^Programa de Mestrado e Doutorado em Fisioterapia, Universidade Cidade de São Paulo (Unicid), São Paulo, Brazil; ^2^Departamento de Fisioterapia, Universidade do Sul de Santa Catarina (Unisul), São Jose, Brazil; ^3^Departamento de Psicologia, Universidade do Oeste de Santa Catarina (Unoesc), Videira, Brazil; ^4^Centro de Investigação Interdisciplinar Egas Moniz (CiiEM), Escola Superior de Saúde Egas Moniz (ESSEM), Almada, Portugal; ^5^Lab Corinthians R9, Sport Club Corinthians Paulista, Sao Paulo, Brazil; ^6^Laboratoire de Recherche BioNR, Universityé du Quebec a Chicoutimi, Saguenay, Canada

## Abstract

**Background:**

It is well known that periods of inactivity generate a loss of muscle strength, a fundamental component of sports performance in soccer. However, little information is available on the decrease in strength levels in professional soccer players after the quarantine lockdown that occurred during the COVID-19 pandemic.

**Aim:**

To compare the isokinetic peak torque profiles of professional soccer players from different teams before and after the quarantine period generated by COVID-19.

**Methods:**

A retrospective observational study was performed using data collected from two different professional elite-level soccer teams just before and immediately after the COVID-19 quarantine period. One team gave individual instructions to its players for conditioning maintenance at home during the quarantine period, while the other team used regular video calls to maintain the player's conditioning status on home training. The main outcomes were the mean peak torque of knee extensors and flexors, from concentric and eccentric contractions of each playing position. *Analysis*. A two-way ANOVA analysis was used to compare peak torque before and after the quarantine period and between both teams' strategies, showing a statistically significant reduction in eccentric knee flexor peak torque from the team that did not have remote monitoring.

**Conclusions:**

Remote monitoring programs are recommended so that athletes are less affected by the deleterious effects of confinement.

## 1. Introduction

The assessment of the peak isokinetic torque is one of the most commonly applied methods of assessing lower extremity muscle strength in soccer [1, 2]. Some authors still use it as a diagnostic tool for the prevention and rehabilitation of sports injuries [3].

It is well known that periods of inactivity generate a loss of muscle strength, a fundamental component of sports performance in soccer [4–10]. However, little information is available on the decrease in strength levels of professional soccer players after the quarantine period applied during COVID-19 pandemic [11–15]. During this period, championships were halted, and players were confined to their homes without proper training and conditioning routines. Different soccer teams used different strategies to avoid physical deconditioning of their players, but few studies have been published so far comparing their results [16].

It is valid to consider motivational aspects in the practice of healthy behaviors during periods of confinement, mostly during the recent lockdown period resulting from COVID-19. Motivation is defined in this study as a multidimensional concept, shaped by individual and contextual factors, responsible for instigating, catalyzing, and maintaining human behavior towards a goal. The phenomenon of motivation for specific behaviors (such as academic activities, health protection, exercise regimes, among others) has been considered an important component in overall adherence to the maintenance of those behaviors. [17].

Therefore, the aim of the present study was to compare the isokinetic peak torque profiles of knee extensors and knee flexors, from concentric and eccentric muscle contractions in elite soccer players before and after the COVID-19 pandemic quarantine period using two different home training strategies, monitored and nonmonitored. Our hypothesis is that soccer players with different home training modalities lose different levels of muscle strength after the COVID-19 quarantine period.

## 2. Methods

### 2.1. Design

This is a retrospective two-arm cohort study based on isokinetic data from Brazilian professional elite-level soccer players evaluated prior to and after the COVID-19 quarantine period. We decided to compare two home training strategies used by each team's athletic trainer during the official lockdown period (6 weeks of quarantine). Also, we compared the results from pre-postquarantine with their isokinetic records pre- and post-interseason period (4 to 6 weeks). Its design was carried out according to the Declaration of Helsinki, and a protocol was fully approved by our University Human Research Ethics Committee with number #4395129. This study followed the Strengthening the Reporting of Observational Studies in Epidemiology (STROBE) Statement and The Improving Healthcare Decisions Task Force (ISPOR Retrospective Databases) guidelines [18].

### 2.2. Participants

The sample size calculation used similar studies concerning isokinetics in soccer [19]. Using an alpha level of 5%, loss of follow-up limited in 10%, and power of 80%, we found that 19 participants in each team/group would be necessary for our study [20]. All calculations used G∗Power Software (University of Düsseldorf, Germany) as used in previous, our already published studies [21].  Table 1 shows the demographic and anthropometric characteristics of the sample before and after the quarantine period.

Two Brazilian soccer teams of professional elite-level accepted to participate in this study allowing their players' isokinetic data to be anonymously analyzed. Both executed a preseason evaluation of all athletes three weeks before the quarantine was officially issued in Brazil, when all local and national championship matches were halted. Also, sports facilities, public gyms, and public parks were closed. Physical training in public spaces is prohibited. Athletes' only option to maintain their physical conditioning was home training. The quarantine for professional sports competitions was lifted six weeks later, and both teams executed a second preseason evaluation of all athletes on the same isokinetic machine, using the same testing protocol with the same physical therapist.

To be included in this study, players had to be able to fully participate in team training sessions and match play at the time of the analysis. Players who had a third-degree knee flexor or knee extensor muscular injury in the past 3 months, had knee surgery in the past 12 months or were currently in treatment from other muscular injuries or illnesses were excluded from our sample. Players with tendon or muscle injury grade I but without symptoms during isokinetic tests were allowed to participate since they do not report pain levels above the designated threshold (see procedures below). Only players who had played in their usual positions for the last year were included in the sample. Once per week, all players were subjected to clinical tests to ascertain they were not infected by the COVID-19 virus.

The purpose, experimental procedures, possible risks, and benefits of the study were explained to the athletes, who provided a written informed consent form to confirm participation in the study. For players under the age of 18, their parents or legal guardians were informed of the risks and signed an informed consent before the investigation enrollment.

### 2.3. Home Training Strategies

The teams used different home training strategies according to recommendations from sports medicine professional associations, issued during the beginning of the pandemic outbreak and recently published. [16].

*Team A* made video calls once per day between each athlete and their athletic trainer to instruct, monitor, and motivate players on their exercise routine.

The training equipment was limited to free-weight dumbbells, kettlebells, and ankle weights. Exercise prescriptions were based on free-weight-bearing and calisthenics activities, two times per day, every day, with each training session lasting one hour. The training plan for the lockdown period was divided into strength exercises (40%), power exercises (20%), and aerobic conditioning (40%). During the quarantine period, participants were requested to eat and drink according to their team's nutritionist prescribed diet, avoid alcoholic beverages, and sleep at least 8 hours per night.

*Team B* gave general instructions for home training from their team's athletic trainer at the beginning of the quarantine. They follow the same training strategy prescription as Team A, using the same kind of equipment. However, players from Team B were not regularly monitored by their trainer during quarantine.

### 2.4. Isokinetic Test Procedure

Participants were requested to eat according to their team's nutritionist's prescribed diet 48 hours preceding the assessment and then refrain from eating and drinking substances other than water one hour before the assessment. All tests were carried out in January, a few weeks before Brazil's regional championship season starts, and players were instructed to refrain from strenuous activities 48 hours before testing.

Upon arrival, the players were provided with an appropriate explanation and demonstration of all procedures. The players informed the most frequently fielded position in the past year's games. Only athletes who played in their usual positions were included in the sample. Positional groupings were goalkeepers (G), defenders (D), wingbacks or “external defenders” (W), midfielders (M), and forwards (F). Dominant leg was defined as the player's preferred kicking leg for a penalty kick. Anthropometric information (body mass in kilograms, height in centimeters) was recorded by the same team nutritionist before the isokinetic test.

All subjects were submitted to a testing protocol following the guidelines of the APTA (American Physical Therapy Association) [4, 22–24] and soccer-specific studies using isokinetic machines [25–27]. The same physiotherapist, with 10 years of experience, performed all the isokinetic tests. All isokinetic tests were performed on both legs, starting with the dominant one.

Participants were positioned on the seat of the isokinetic dynamometer and executed 10 repetitions of concentric knee flexion and extension, both with a velocity of 90 degrees per second and a range of motion of 100 degrees for warming up purposes (Borg up to 5, VAS up to 1, or test interrupted), followed by a rest period of 120 seconds. The warm-up on the isokinetic machine was chosen to improve specificity and familiarization with the following test [24]. The athlete then performed five concentric repetitions of knee flexion and extension at 60 degrees per second for familiarization with the exercise velocity, followed by another rest period of 120 seconds. Then they performed three concentric repetitions of knee flexion and extension (velocity: 60 degrees per second and range of motion: 100 degrees) with maximum effort (Borg 10), receiving continuously, the standardized verbal encouragement, “Faster.” The presence of pain equal or superior to 05 on VAS interrupted the test, canceling it and excluding the subject from the sample. The repetition with the higher peak torque value of all three repetitions was used for statistical analysis.

The eccentric test was performed at 60 degrees per second and with a range of motion of 100 degrees. The subject executed 5 repetitions of warm-up and familiarization followed by 3 repetitions at maximum effort (Borg 10), receiving constantly the standardized verbal encouragement, “Hold it.” The presence of pain equal or superior to 05 on VAS interrupted the test, canceling it and excluding the subject from the sample. Between each set of exercises, subjects had 120 seconds to rest. Between each limb's test, participants had 120 seconds to rest.

For data collection, the following instruments were used: an isokinetic dynamometer (Cybex-CSMI, model HumacNorm 2009, Stoughton, Massachusetts, USA) with a signal acquisition rate of 500 Hz. In order to improve players' test understanding, we used a modified 10-points Borg scale (BORG) for perceived exertion, where zero was no strength effort and 10 the maximum strength effort possible. We used a visual analog pain scale (VAS), where zero was “no pain” and 10 was “worst pain imaginable” [28]. For data storage and processing, we used a MacBook Pro Notebook (Cupertino, California, USA) equipped with Microsoft Office software package for Mac (version 2011, Redmond, Washington, USA) and the Statistical Package for Social Sciences (SPSS) from IBM (Armonk, NY, USA).

### 2.5. Data Analysis

Concentric peak torque (CPT) and eccentric peak torque (EPT) of knee extensors and flexors were extracted from the isokinetic machine by its manufacturer's dedicated software (HumacNorm 2009, CSMi Inc, Boston, US) and normalized by each subject's body mass.

The descriptive analysis of the data was composed of simple and relative frequencies of the variables: concentric peak torque (CPT), eccentric peak torque (EPT), conventional H/Q ratio (flexors CPT divided by extensor CPT), and functional H/Q ratio (flexors EPT divided by extensor CPT).

The data's normality was confirmed using visual inspection and the *Kolmogorov–Smirnov* tests. Homogeneity of variance was assessed via *Levene's test*. Data with normal distribution were subjected to a two-way analysis of variance (ANOVA). Posthoc analysis used the Bonferroni test, adjusted for multiple comparisons. Size effects were measured between subjects partial-eta square (*η*^2^). The magnitude was categorized as small (0.01), moderate (0.06), and large (0.14), respectively. All data were processed using SPSS v.20 (IBM, Chicago, IL, USA) with statistical significance set at alpha level *p* < 0.05.

## 3. Results

Anthropometric characteristics for all players are provided in Table 1. Results indicated that our sample had no significant differences in body mass and height between intra- or inter-group.

Table 2 shows inferential analysis of team A strength values before (pre) and after (post) the quarantine period. No significant statistical difference was found for isokinetic peak torque of knee extensors or flexors, from concentric or eccentric contraction modes.

Table 3 shows inferential analysis of team B strength values before (pre) and after (post) the quarantine period. A significant statistical difference was found for isokinetic peak torque of knee flexors eccentric contraction mode (*p* < 0.01). Cohen's partial-eta square indicates a large (*d* = 2. 31) magnitude effect. Statistical differences with large size effects (*d* = 3.44) were also found on functional H/Q ratios.

## 4. Discussion

Our study investigated the effects of the quarantine-lockdown period due to the COVID-19 pandemic on the strength (isokinetic torque) of professional soccer players on two different Brazilian professional soccer teams. Our main findings show that there was no significant reduction in torque of knee extensors or flexors in team A. However, in team B, there was a significant reduction in the eccentric strength of knee flexors. Both teams sought to avoid the harmful effects of the detraining period by using different strategies. Team A monitored the training routine through video calls, while team B did not implement the training routine monitoring strategy. Therefore, monitoring training can be a strategy to increase players' adherence to training routines and avoid the deleterious effects of long periods of confinement as in COVID-19 lockdown.

The COVID-19 pandemic and its preventive measures significantly impacted the physical and functional capacities of amateur and professional athletes [29, 30]. A study conducted with professional soccer players found a significant drop in relative distance, maximum speed, acceleration, and deceleration in soccer athletes [31]. Another similar study verified a significant loss in the aerobic capacity of soccer players, even though they had performed a supervised home exercise program [15]. This drop ranged from 10 to 17%, verified by the Yo-Yo test (level 2) in both studies.

Another study investigated values of 1 RM in squat, jump height, and sprint performance in female soccer players and found no significant drop after 12 weeks, probably due to high adherence to the home training program [32]. On the other hand, a significant reduction in knee flexor strength was detected after 25 days of confinement at home by other researchers [11]. The difference between the results can be explained by the fact that eccentric strength is more susceptible to decreases compared to other neuromuscular factors. Our results supported that conclusion.

Researchers expected that the restrictions caused by COVID-19 would lead to training interruption and, consequently, to physiological detraining during the pandemic, highlighting the potential effect of the confinement period on neuromuscular characteristics [33, 34]. During the lockdown period, there were severe training restrictions and a substantial reduction in the overall level of physical activity for almost all individuals. However, our results demonstrate that there was no such significant reduction in the strength level (isokinetic torque) in team A, as reported in previous research on detraining [33, 35]. This can be justified as players of both teams did not completely interrupt their training routine, which may have contributed to attenuating the effect of detraining.

Similar to our findings, a previous study demonstrated that a group of semiprofessional soccer players had their knee flexor strength reduced after 25 days of confinement caused by COVID-19. However, these findings are not directly comparable, since the measurement in that study was made through the Nordic flexion exercise, which differs from our study, even though both tests are capable to assess the eccentric strength of the knee flexors. Even though eccentric strength has been shown to be more susceptible to decline compared to other neuromuscular factors [36].

As eccentric contractions of knee flexors are directly related to functional H/Q ratios and this index is regularly used as a predictor of muscular injury risk, the decline of knee flexor torque becomes an important characteristic to be avoided during a quarantine period. Our results have shown that the functional H/Q ratio was largely affected on team B, in accordance with its own decline in eccentric flexor peak torque. In this case, the absence of enough motivational support from the coaches cannot only negatively the players' strength but also increase their muscular injury risk.

In the context of sport, the time period resulting from the suspension of sports practice, associated with cognitive anxiety and self-determined motivations for returning to competition, can impair athletic performance and increase the risk of future injuries [37]. It is noteworthy that differences in perceived stress, emotional state, and personality variables were related between athletes less motivated to train compared to athletes who do not report changes in their motivational states, which may be an additional factor to the difference between teams A and B in our study [38].

Overall, our results are in accordance with previous research, which suggests the need for motivational practices from coaches to increase adherence towards their teams training strategy, thus avoiding athletic performance decline during exceptional contexts [38]. As seen from a similar study [38], suggesting that athletes, even receiving emotional support with training routines programmed with schedules and applications, still reported the need for more support from their coaches and trainers.

## 5. Conclusions

Our results showed that the eccentric strength of knee flexors was significantly reduced in the group that did not get frequent monitoring of the training regimen during the confinement period. Remote monitoring programs are recommended so that athletes are less affected by the deleterious effects of confinement. The increase in cognitive-behavioral interventions can be an efficient strategy to collaborate with motivational and psychological aspects and return to sport.

## Figures and Tables

**Table 1:** Demographic and anthropometric characteristics of the sample before and after the quarantine period (means ± standard deviations).

	Team A	Team B
*Prequarantine*		
Participants (*n*)	26	23
Age (years)	26.15 ± 5.12	26.47 ± 5.34
Height (cm)	181.46 ± 6.92	180.60 ± 6.59
Body mass (kg)	77.03 ± 7.99	76.08 ± 8.43
BMI (kg/m^2^)	23.39 ± 0.17	23.33 ± 0.19

*Postquarantine*		
Participants (*n*)	26 ± 23	
Age (years)	26.15 ± 5.12	26.47 ± 5.34
Height (cm)	181.46 ± 6.92	180.60 ± 6.59
Body mass (kg)	76.19 ± 6.71	74.55 ± 7.93
BMI (kg/m^2^)	23.14 ± 0.14	22.86 ± 0.18

**Table 2:** Team A strength values before (pre) and after (post) quarantine period.

	Pre		Post		Mean Difference		95% CI		*p*†	*d*‡
*Knee extensors*	M	±SD	M	±SD	M	±SD				
Concentric peak torque	242.70	43.21	244.04	38.09	2.19	54.93	−19.99	24.37	0.84	NA
Eccentric peak torque	304.41	47.29	302.04	54.82	3.84	59.77	−20.29	27.98	0.74	NA

*Knee flexors*										
Concentric peak torque	142.62	25.30	154.04	27.01	−9.53	34.86	−23.62	4.54	0.17	NA
Eccentric peak torque	179.79	27.06	175.91	31.06	7.50	44.29	−10.38	25.38	0.39	NA

*HQ ratios*										
Conventional	0.59	0.06	0.63	0.05	0.04	0.03	−12.66	9.31	0.91	NA
Functional	0.74	0.03	0.72	0.04	−0.02	0.01	−8.11	6.14	0.72	NA

Mean (M); standard deviation (SD); confidence interval of 95% (CI); not applicable (NA); ^∗^difference statistically significant (*p* < 0.05); † alfa level *p* < 0.05; and ‡ cohen *d* effect size.

**Table 3:** Team B strength values before (pre) and after (post) quarantine period.

	Pre		Post		Mean Difference		95% CI		*p*†	*d*‡
*Knee extensors*	M	±SD	M	±SD	M	±SD				
Concentric peak torque	241.91	46.05	238.29	47.35	3.62	67.03	−24.68	31.93	0.79	NA
Eccentric peak torque	294.29	61.55	293.04	44.11	1.25	74.17	−30.07	32.57	0.93	NA

*Knee flexors*										
Concentric peak torque	148.75	24.91	144.75	24.34	4.00	34.77	−10.68	18.68	0.57	NA
Eccentric peak torque	**294.37**	61.60	**185.75**	27.10	**108.62**	72.72	77.91	139.33	**<0.01**	**2.31**

*HQ ratios*										
Conventional	0.61	0.12	0.61	0.05	−0.01	0.03	−7.23	15.44	0.81	NA
Functional	**1.22**	0.36	**0.78**	0.10	**−0.44**	0.01	11.27	21.48	**<0.01**	**3.44**

Mean (M); standard deviation (SD); confidence interval of 95% (CI); not applicable (NA); ^∗^difference statistically significant (*p* < 0.05); † alfa level *p* < 0.05; and ‡ cohen *d* effect size. Bold measures showed statistical significancy.

## Data Availability

Additional data from this study were not allowed to be shared by participant institutions.

## Additional Points

(i) It is valid to consider motivational aspects in the practice of healthy behaviors during periods of confinement, mostly during the recent lockdown period resulting from COVID-19 when all competitive sports practice was halted. (ii) Two Brazilian soccer teams of professional elite-level accepted to participate in this study allowing their player's isokinetic strength data to be anonymously analyzed just before and after the 6 weeks lockdown period. Team A players received daily video calls while team B players received a training regime plan but without monitoring. (iii) The results showed that team B had a significant reduction in the strength of their knee flexors (the most injured muscle in soccer), increasing the injury risk factor of those players. This suggests that monitoring and motivational aspects worked to prevent strength and performance levels from decreasing in team A.
